# Preoperative Three-Dimensional Lung Simulation Before Thoracoscopic Anatomical Segmentectomy for Lung Cancer: A Systematic Review and Meta-Analysis

**DOI:** 10.3389/fsurg.2022.856293

**Published:** 2022-03-31

**Authors:** Zhongtian Xiang, Bo Wu, Xiang Zhang, Nan Feng, Yiping Wei, Jianjun Xu, Wenxiong Zhang

**Affiliations:** Department of Thoracic Surgery, The Second Affiliated Hospital of Nanchang University, Nanchang, China

**Keywords:** segmentectomy, lung cancer, systematic review, meta-analysis, three-dimensional lung simulation

## Abstract

**Background:**

Whether the utilization of preoperative three-dimensional (3D) lung simulation can improve the outcomes of segmentectomy for lung cancer (LC) is still controversial. Our meta-analysis was performed to compare preoperative 3D lung simulation with non-3D procedures in terms of perioperative outcomes.

**Methods:**

Seven databases (Embase, Ovid Medline, ScienceDirect, PubMed, Web of Science, Cochrane Library, and Scopus) were searched for eligible articles. Intraoperative outcomes (conversion, operative time, etc.), postoperative indicators (postoperative hospital stay, total number of complications, etc.) and postoperative complications were endpoints.

**Results:**

After applying predefined inclusion criteria, we included 8 studies and 989 patients (3D group: 552 patients; non-3D group: 437 patients) in our meta-analysis. The results of the meta-analysis showed that preoperative 3D lung simulation could significantly decrease the blood loss (mean difference [MD]: −16.21 [−24.95 to −7.47]ml, *p* = 0.0003), operative time (MD: −13.03 [−25.56 to −0.50]ml, *p* = 0.04), conversion rate (conversion from segmentectomy to thoracotomy or lobectomy) (MD: 0.12 [0.03–0.48], *p* = 0.003), postoperative hospital stay (MD: −0.25 [−0.46 to 0.04]days, *p* = 0.02) and total number of complications (MD: 0.59 [0.43–0.82], *p* = 0.001) compared with non-3D procedures. The number of resected lymph nodes (LNs), postoperative drainage time, postoperative forced expiratory volume in the first second (postoperative FEV1) and postoperative drainage volume were similar in the two groups. Arrhythmia (5.30%), pulmonary air leakage (2.72%), atrial fibrillation (2.20%), pulmonary infection (2.04%), and pneumonia (1.73%) were the top 5 postoperative complications in the 3D group.

**Conclusions:**

Preoperative 3D lung simulation was better than non-3D procedures in segmentectomy for LC, with better intraoperative and postoperative outcomes. However, our results should be confirmed in larger prospective randomized controlled trials.

**Systematic Review Registration:**

PROSPERO, identifier: CRD42021275020.

## Introduction

Lung cancer (LC) remains the leading cause of cancer death, and it is the cancer with the highest incidence (1). In recent years, the detection rate of early non-small-cell lung cancer (NSCLC) has increased significantly with the introduction of thin-section and low-dose computed tomography (2). The application of segmentectomy, which is associated with less trauma, fewer complications, and less pain for patients than thin-section and low-dose computed tomography, is more conducive for patients with non-small-cell lung cancer (3). 3D computed tomography of lung segments can ensure the position of the lung tumor within the anatomical segment and assist in the prediction of surgical margins (4). However, whether preoperative 3D lung simulation is better than non-3D procedures in segmentectomy for lung cancer remains controversial.

Whether preoperative 3D lung simulation is better than non-3D procedures in segmentectomy for LC has not yet been confirmed by guidelines (5). Liu et al., Chen et al., and Xu et al. suggested that preoperative 3D simulations for the assessment of pulmonary vessel and bronchi branching patterns could improve surgical accuracy and safety (6–8). Similarly, Hu et al., Xue et al., She et al., and Qiu et al. all reported that preoperative 3D lung simulation shortened the operation time and reduced intraoperative blood loss and intraoperative and postoperative complications, especially in patients with stage IA NSCLC (9–12). However, Wu et al. suggested that conventional segmentectomy can obviously reduce the time of operation and can protect the lung from air leakage, although a sufficient resection margin and lymph node dissection might be ensured by preoperative 3D lung simulation (13).

To verify the reliability and safety of this method, we compared the intraoperative and postoperative indicators of the included patients for whom preoperative 3D lung simulation was performed with those of patients for whom it was not performed.

## Materials and Methods

This meta-analysis followed the guidelines of the Preferred Reporting Items for Systematic Reviews and Meta-Analyses (PRISMA) (Supplementary Table 1) (14). (PROSPERO Registration: CRD42021275020).

### Search Strategy

Seven databases (Embase, Cochrane Library, Scopus, PubMed, Ovid Medline, Web of Science, and Science Direct) were searched from their inception date to the 26th of August 2021. We used the MeSH terms “three-dimensional” and “segmentectomy” as follows. The gray literature, abstracts and bibliographies were investigated for additional qualified reports. The comprehensive retrieval scheme is shown in Supplementary Table 2.

### Selection Criteria

Inclusion criteria:

(1) Sample: patients with lung cancer who underwent segmentectomy.
(2) Intervention and comparison: preoperative 3D lung simulation compared with non-3D procedures.
(3) Outcomes: intraoperative outcomes (blood loss, operative time, conversion, number of resected LNs) and hospitalization and follow-up outcomes (postoperative drainage time, total number of complications, postoperative drainage volume, postoperative FEV1 and postoperative hospital stay).
(4) Study design: RCTs and cohort studies.

Only studies of human subjects were included. If the institution published repeated trials and patient cohorts were included, we selected the most recent complete study for evaluation. Commentaries, expert recommendations, editorials, and conference abstracts were excluded. Because of the possibility of the duplication of results or publication bias, we also excluded review articles.

### Data Extraction

We constructed a standardized data extraction table in which to record the following data from the included studies: publication year, first author, nation, study period, patient characteristics (sex, age), tumor characteristics (location, histology), intraoperative outcomes (blood loss, operative time, conversion, number of resected LNs), and postoperative outcomes (postoperative drainage time, total number of complications, postoperative drainage volume, postoperative FEV1 and postoperative hospital stay). When a study had both propensity-matched and non-propensity-matched data, we chose the propensity-matched data for the included analysis. The extraction of data from all included studies was performed independently by 2 investigators. In the case of disagreement, a third coauthor's opinion was sought, and the disagreement was resolved by consensus.

### Quality Assessment

We utilized the Newcastle–Ottawa Scale (NOS) to evaluate the quality of 8 searched studies. This scale contained 3 items: comparability, outcome, and selection. Each study was scored based on the above three factors on a scale of 0–9 (allocated as stars), and studies with scores ≥7 were defined as being of high quality, those with scores ≤ 4 points low quality, and those with scores ≥5 but ≤ 6 points medium quality (15). The 5-point Jadad scale was applied to evaluate the quality of RCTs. Three items were included in the scale: the masking, randomization, and accountability of included patients. Scores ≥3 points indicated high quality (16).

A Grades of Recommendations Assessment, Development and Evaluation (GRADE) table was applied to assess the evidence level of those results. This standard included 5 items: indirectness, risk of bias, publication bias, indirectness imprecision and inconsistency. Studies were evaluated as having a high, moderate, low or very low level of evidence (17).

### The Analysis of Statistical Data

We performed statistical analysis using STATA 12.0 and Review Manager Version 5.3 (Software Update, Cochrane Collaboration, UK, Oxford). Additionally, researchers calculated the corresponding 95% confidence interval (95% CI) and computed the risk ratio (RR) for dichotomous outcomes. For continuous outcomes, the mean difference (MD) is presented. The standardized mean difference (SMD) is presented if various tables were required to measure similar basic constructions. The corresponding 95% CIs were determined for all outcomes. To evaluate heterogeneity, we mainly used the *I*^2^ statistic and χ^2^ test. We also used a random-effects model for notable heterogeneity (*I*^2^ > 50% or *p* < 0.1). Otherwise, a relatively fixed-effects model was chosen. Egger's (18) and Begg's (19) tests were applied for the assessment of publication bias. In particular, differences with *P*-values < 0.05 were defined as significant.

## Results

### Search Results and the Quality of the Included Studies

**Figure 1:**
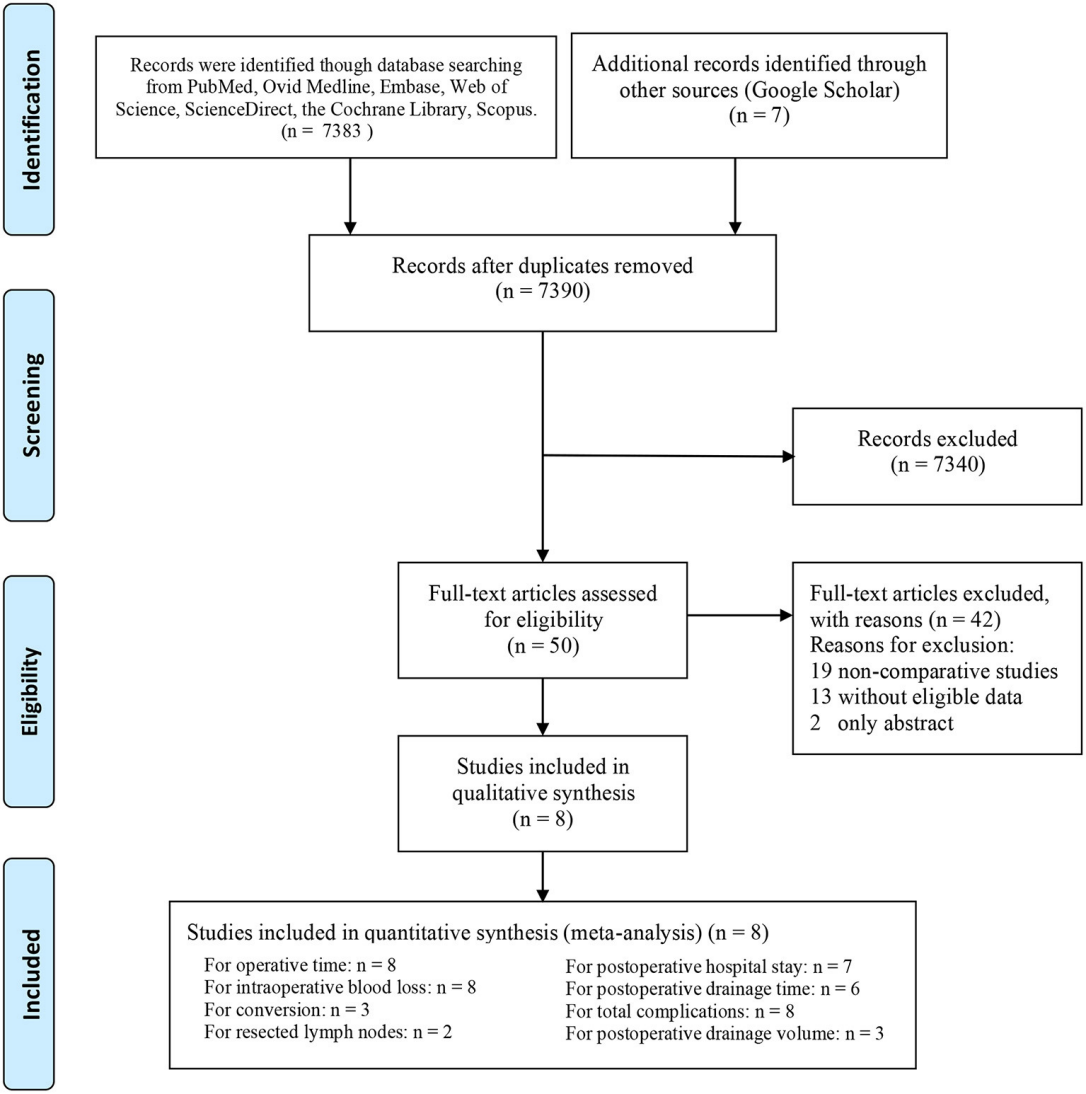
Flow diagram of the research selection process.

Ultimately, 8 articles involving 989 patients (3D group: 552, non-3D group: 437) were included for assessment (6–13) (Figure 1). The baseline characteristics are shown in Table 1. As determined by the NOS and Jadad scale, all studies were of medium-high quality (Table 2). The quality evidence of the outcomes was low and very low, in line with the GRADE list (Supplementary Table 3).

**Table 1:** Summary of the baseline characteristics of the included studies.

**Study**	**Nation**	**Period (year)**	**Groups**	**Patients**	**Sex (M/F)**	**Age (Mean, year)**	**Lesion location (lobes)**						**Histology(n)**						**Follow up (months)**
							**Right**			**Left**			**Atypical adenomatous hyperplasia**	**Adenocarcinoma** **in situ**	**Minimally** **invasive** **adenocarcinoma**	**Invasive** **adenocarcinoma**	**Microinvasive** **adenocarcinoma**	**0thers**	
							**Upper**	**Middle**	**Lower**	**Upper**	**Lower**								
2021	Wu et al. (13)	China	2020.1–2020.9	3D	55	20/35	52.9	12	–	14	16	13	–	10	–	4	41	–	–
				Non-3D	55	23/32	53.1	13	–	13	16	13	–	3	–	6	46	–	
2021	Hu et al. (9)^a^	China	2019.1–2020.8	3D	30	14/16	61.3	10	–	3	14	3	1	3	23	4	–	5	–
				Non-3D	35	16/19	62.7	11	–	4	20		5	2	5	22	5	–	6
2020	Chen et al. (7) RCT	China	2016.3–2018.9	3D	51	21/30	60.7	16	–	17	11	7	–	–	–	–	–	–	–
				Non-3D	38	14/24	61.6	11	–	14	8		5	–	–	–	–	–	–
2020	Qiu et al. (12)	China	2017.4–2019.5	3D-model	31	6/25	54.5		16		15		2	12	–	19	16	–	3.0
				3D-RS^a^	131	42/89	54.4		54			77		4	35	–	87	48	–
				Non-3D	136	41/42	54.4		57			79		5	23	–	89	44	–
2019	Liu et al. (6)	China	2017.10–2018.8	3D-CT	39	13/26	60.6	12	–	11	10		6			34		5	–
				3D-printing	32	13/19	61.4	14	–	4	11		3			27			5
				Non-3D	53	19/34	62.1	18	–	11	13		11			45			8
2019	Xu et al. (8)	China	2017.7–2018.11	3D	96	37/59	50.4	38	–	13	26	19	–	–	–	22	69	–	–
				Non-3D	37	16/21	53.3	18	–	1	12	6	–	–	–	9	26	–	
2018	Xue et al. (10)	China	2016.5–2017.2	3D	36	9/27	53.0	12	–	6	13	5	–	5	–	8	23	–	17.5
				Non-3D	32	12/20	51.6	10	–	5	12	5	–	5	–	9	18	–	
2018	She et al. (11)	China	2014.1–2017.5	3D	51	21/30	59.3	13	–	15	13	10	5	23	17	–	–	6	12.0
				Non-3D	51	24/27	58.5	12	–	14	13	13	3	20	19	–	–	9	

*3D, Three-dimensional; 3D-RS, 3D-reconstruction; M/F, male/female*.

a *The number of patients in Histology (n) were inconsistent with the number of patients in Groups because some patients may have multiple lesions*.

**Table 2:** Methodological quality assessments of the included studies.

**Study**		**Randomization**	**Masking**	**Accountability of all patients**	**Selection**				**Comparability^**d**^**	**Outcome**			**Total score**
				**Exposed cohort^**a**^**	**Non-exposed cohort^**b**^**	**Ascertainment of exposure**	**Outcome of interest^**c**^**		**Assessment of outcome**	**Length of follow-up^**e**^**	**Adequacy of follow-up**	
2021	Wu et al. (13)				*	*	*	*	**	*			7
2021	Hu et al. (9)				*	*	*		**	*			6
2020	Chen et al. (7) RCT	**	**	*									5
2020	Qiu et al. (12)				*		*	*	**	*	*	*	8
2019	Liu et al. (6)				*		*	*	**	*			6
2019	Xu et al. (8)				*	*	*	*	**	*			7
2018	Xue et al. (10)				*	*	*	*	**	*	*	*	9
2018	She et al. (11)				*	*	*	*	**	*	*	*	9

a *Representativeness of the exposed cohort;*

b *Selection of the non-exposed cohort;*

c *Demonstration that outcome of interest was not present at start of study;*

d *Comparability of cohorts on the basis of the design or analysis;*

e *Was follow-up long enough for outcomes to occur*.

### Intraoperative Outcomes

**Figure 2:**
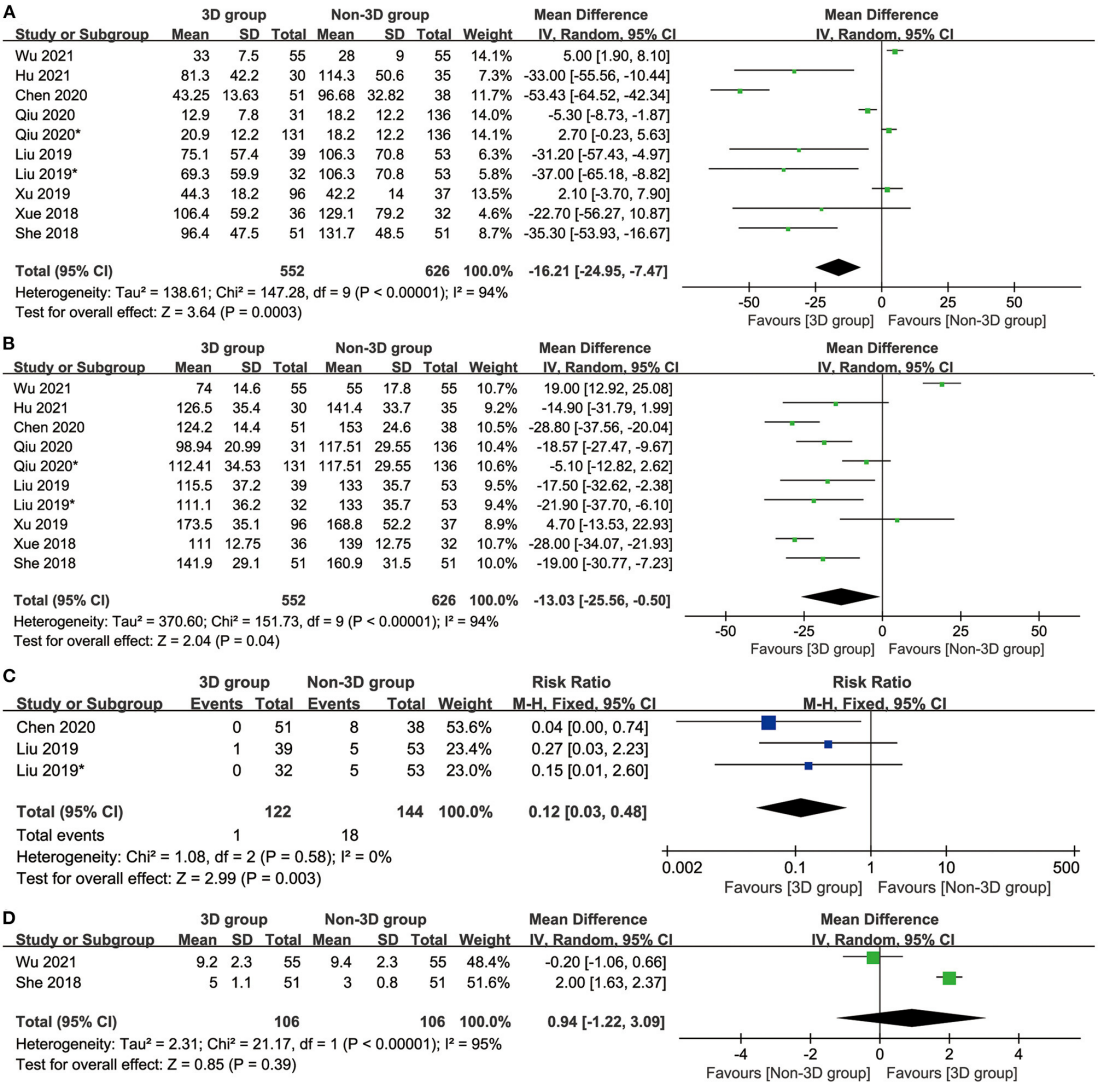
Forest diagrams of intraoperative indicators: blood loss **(A)**, operative time **(B)**, conversion **(C)**, resected LNs **(D)**.

No significant difference was reported between the two groups in the number of resected LNs (MD: 0.94 [−1.22 to 3.09], p = 0.39, Figure 2D).

The 3D group had less blood loss (mean difference [MD]: −16.21 [−24.95 to −7.47] ml, p = 0.0003, Figure 2A), a shorter operative time (MD: −13.03 [−25.56 to −0.50] ml, p = 0.04, Figure 2B) and a lower conversion rate (conversion from segmentectomy to thoracotomy or lobectomy) (mean difference: 0.12 [0.03–0.48], p = 0.003, Figure 2C) than the non-3D group.

### Hospitalization and Postoperative Indicators

**Figure 3:**
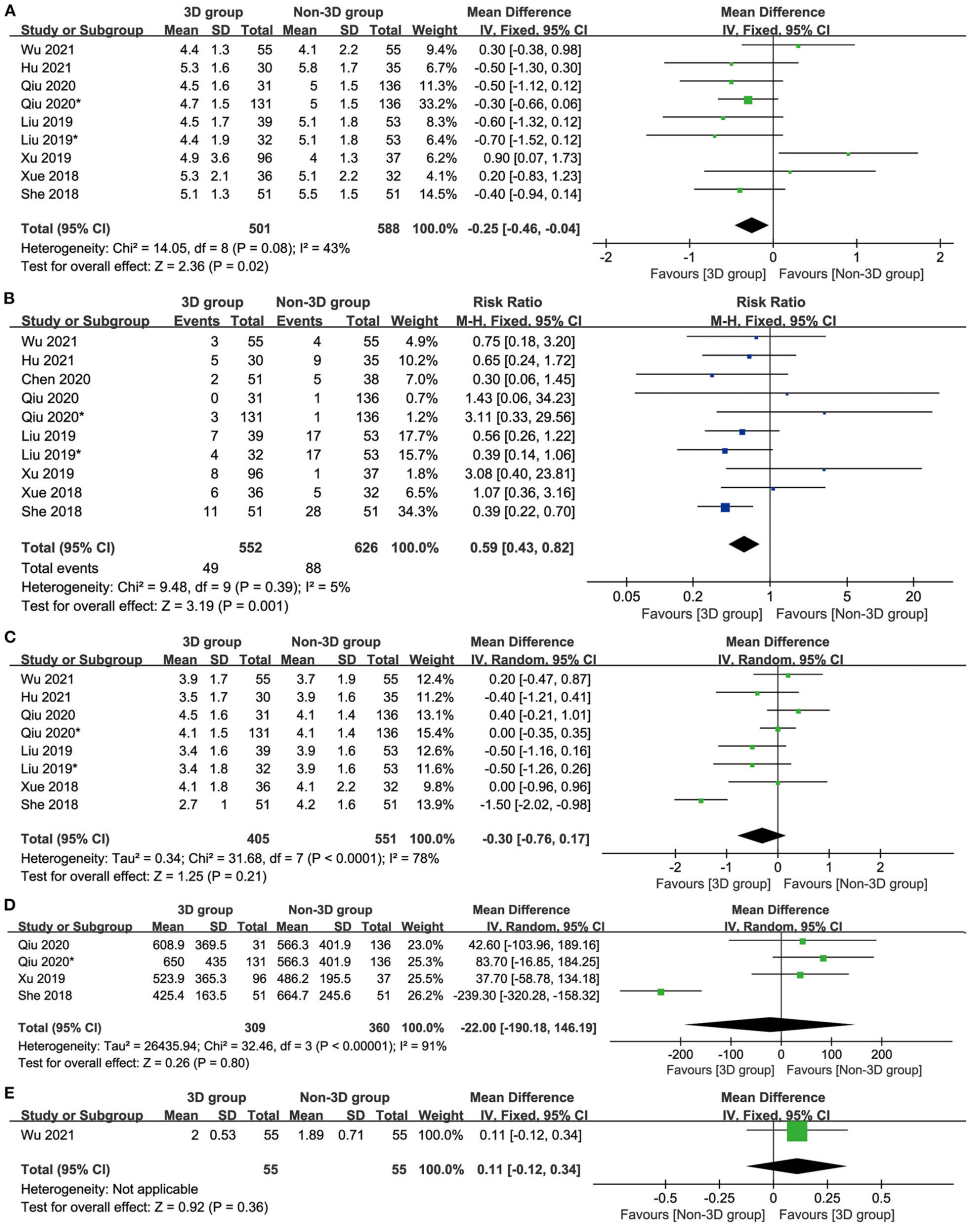
Forest plots of hospitalization indicators: postoperative hospital stay **(A)**, complications **(B)**, postoperative drainage time **(C)**, postoperative drainage volume **(D)**, and postoperative Forced Expiratory Volume in the first second **(E)**.

The 3D group had a shorter postoperative hospital stay (MD: −0.25 [−0.46 to −0.04] days, p = 0.02, Figure 3A) and a lower total number of complications (MD: 0.59 [0.43–0.82], p = 0.001, Figure 3B) than the non-3D group. The postoperative drainage time (MD: −0.30 [0.76–0.17], p = 0.21, Figure 3C), postoperative drainage volume (MD: −22.00 [−190.18–146.19], p = 0.80, Figure 3D) and postoperative FEV1 (MD: 0.11 [−0.12 to 0.34], p = 0.36, Figure 3E) were similar between these two groups.

### Complications

The total number of complications was similar between the two groups. In the 3D group, the top 5 complications were arrhythmia (5.30%), pulmonary air leakage (2.72%), atrial fibrillation (2.20%), pulmonary infection (2.04%) and pneumonia (1.73%). In the non-3D group, the top 5 complications were arrhythmia (8.87%), hemoptysis (8.25%), pulmonary air leakage (5.49%), pulmonary infection (2.67%), and atelectasis (2.26%) (Table 3).

**Table 3:** Postoperative complications.

**Postoperative complications**	**Studies involved**	**3D group**		**Non-3D group**		**Total incidence**	**Differences (95% CI)**	***I*^2^ (%)**	**P**
		**Event/total**	**%**	**Event/total**	**%**				
Pulmonary air leakage	8	15/552	2.72%	24/437	5.49%	3.94%	0.53 [0.28–1.01]	0	0.05
Pneumonia	6	7/405	1.73%	8/362	2.21%	1.96%	0.83 [0.33–2.08]	0	0.69
Atelectasis	5	6/371	1.62%	7/310	2.26%	1.91%	0.72 [0.26–2.01]	0	0.53
Hemoptysis	4	3/207	1.45%	16/194	8.25%	4.74%	0.19 [0.06–0.58]	0	0.004
Arrhythmia	3	7/132	5.30%	11/124	8.87%	7.03%	0.67 [0.28–1.60]	0	0.37
Atrial fibrillation	2	2/91	2.20%	0/87	0.00%	11.23%	2.83 [0.30–26.68]	0	0.36
Cerebral infarction	2	2/147	1.36%	1/75	1.33%	1.35%	0.91 [0.12–7.17]	0	0.93
Pulmonary infection	2	3/147	2.04%	2/75	2.67%	2.25%	0.75 [0.13–4.10]	0	0.74
Liquid pneumothorax	1	1/96	1.04%	0/37	0.00%	0.75%	1.18 [0.05–28.22]	–	0.92
Postoperative hemothorax	1	1/96	1.04%	0/37	0.00%	0.75%	1.18 [0.05–28.22]	–	0.92
Pleural effusion	1	1/96	1.04%	0/37	0.00%	0.75%	1.18 [0.05–28.22]	–	0.92

*CI, Confidence interval; COPD, Chronic obstructive pulmonary disease*.

### Sensitivity Analysis

In the analysis of blood loss (Supplementary Figure 1A), postoperative drainage time (Supplementary Figure 1B) and postoperative hospital day (Supplementary Figure 1C), we found significant heterogeneity. The results of the sensitivity analysis suggested that the omission of each study had little influence on the reliability of the results.

### Publication Bias

We analyzed the postoperative drainage time (Supplementary Figure 2A), operative time (Supplementary Figure 2B) and postoperative hospital day (Supplementary Figure 2C), and no evidence of publication bias was identified.

## Discussion

Currently, the leading cause of cancer-related mortality worldwide is still lung cancer (20). According to global epidemiological data, over 2 million individuals were affected by lung cancer in 2018 (21). At present, segmentectomy in combination with mediastinal lymph node (LN) dissection or sampling is still one of the main treatment options for patients with stage I non-small-cell lung cancer (NSCLC) (22–24). 3D imaging provides a stereoscopic view, and the visual information obtained through binocular visualization allows precise 3D preoperative planification (25). However, large-sample studies demonstrating the safety and efficacy of 3D system use are still lacking. This study is the first meta-analysis of preoperative 3D lung simulation compared with non-3D procedures during segmentectomy for lung cancer. Our results revealed that the 3D group had notably less blood loss, a shorter operative time, a lower conversion rate, a shorter postoperative hospital stay and a lower total number of complications than the non-3D group. Arrhythmia, pulmonary air leakage, atrial fibrillation, pulmonary infection and pneumonia were top 5 postoperative complications in the 3D group. The number of resected lymph nodes (LNs), postoperative drainage time, postoperative forced expiratory volume in the first second (post-op FEV1) and postoperative drainage volume were similar in the two groups.

For intraoperative outcomes, the patients in the 3D group showed an obvious improvement in blood loss, operative time, and conversion rate (conversion from segmentectomy to thoracotomy or lobectomy) compared with patients in the non-3D group. With the latest developments in 3D lung simulation imaging, the quality of imaging in a 3D system is similar to that in stereo vision. Because the 3D structure of the lung was examined adequately before surgery, the risk of bleeding during surgery was reduced. The surgeon obtained a better understanding of the anatomy of the nodule by watching the preoperative 3D lung simulation, which may have led to the shorter operation time. Similarly, with preoperative 3D lung simulation scanning, conversion to lobectomy was less likely. However, there were no differences in the number resected LNs between these two groups. Jiao et al. found that their 3D group had results that were similar to those of their traditional 2D procedure group (26). Regarding the number of resected lymph nodes, preoperative 3D lung simulation provided a better understanding of the position of LNs than traditional 2D procedures, thus increasing the number of resected LNs.

For postoperative outcomes, the postoperative hospital stay and total number of complications in the 3D group were better than those of the non-3D group (27). However, Hu W et al. found that the postoperative hospital stay and total number of complications such as pneumonia, hemoptysis, arrhythmia, and pulmonary air leakage were not significantly different between the two groups (9). Postoperative complications are often caused by multiple factors, usually intraoperative conditions, postoperative patient care and primary underlying disease. A study with a larger sample is needed to further confirm and clarify these findings. For the postoperative drainage time, postoperative FEV1 and postoperative drainage volume, we found little difference between the two groups. However, Xu et al. reported that their 3D group had notably different postoperative drainage times compared with their 2D group (28). On the one hand, the difference between the two studies might caused by differences in the condition of the patients, for example, the basic physical condition of the patients and the patients' other underlying diseases, as well as differences in clinical nursing practices (29). It is worth mentioning that the proficiency of the surgeons who performed segmentectomy for lung cancer also may be partly responsible for the difference between the two studies. On the other hand, preoperative 3D lung simulation improved preoperative preparation, which therefore may have reduced the risks of difficulties and accidents during the operation (30), resulting in the 3D group having better postoperative drainage times.

There are still many shortcomings in our research. First, the sample size was too small to allow credible conclusions to be drawn, and the number of patients was generally <100 in the 3D group (31). Second, only one study was a randomized controlled trial, and the remaining studies were cohort studies of lower quality than the RCT. In the future, more randomized controlled trials and studies with larger sample sizes are needed. Third, none of these 8 studies reported survival data, which is an important clinical result. However, this situation is likely due to the development of preoperative 3D lung simulation in recent years, which limits the availability of longer-term survival data at present and demands the accumulation of time and efforts. Fourth, all studies and patients were from China, which may lead to racial bias in the results, which may not be applicable to other regions and populations. Last, the indicators monitored were not consistent among the studies. Some studies focused on intraoperative indicators, but other studies tended to analyze postoperative indicators. A broad and unified standard is necessary to standardize and evaluate the two surgical methods.

Overall, preoperative 3D lung simulation showed a beneficial improvement in perioperative outcomes and postoperative outcomes compared with non-3D procedures for segmentectomy with lung cancer. The top 5 common complications in the 3D group were arrhythmia, pulmonary air leakage, atrial fibrillation, pulmonary infection and pneumonia. Because of the above limitations, our results should be verified in large-sample randomized controlled trials. Preoperative 3D lung simulation improves surgical accuracy and safety and is worthy of clinical promotion.

## Data Availability Statement

The original contributions presented in the study are included in the article/Supplementary Material, further inquiries can be directed to the corresponding author/s.

## Author Contributions

ZX and WZ had full access to all of the data in the manuscript and takes responsibility for the integrity of the data and the accuracy of the data analysis. ZX, JX, and WZ: drafting of the manuscript. ZX, BW, XZ, NF, YW, and WZ: critical revision of the manuscript for important intellectual content. ZX, BW, XZ, and YW: statistical analysis. ZX and WZ: supervision. All authors: concept, design, acquisition, analysis, or interpretation of data. All authors contributed to the article and approved the submitted version.

## Funding

This study was supported by Natural Science Foundation of Jiangxi Province (Grant number: 20212BAB206050), Science and technology planning project of Health Commission of Jiangxi Province (Grant number: 202110045) and Science and technology planning project of Jiangxi Administration of traditional Chinese Medicine (Grant number: 2020B0108). The funding had no role in the design and conduct of the study; collection, management, analysis, and interpretation of the data; preparation, review, or approval of the manuscript; and decision to submit the manuscript for publication.

## Conflict of Interest

The authors declare that the research was conducted in the absence of any commercial or financial relationships that could be construed as a potential conflict of interest.

## Publisher's Note

All claims expressed in this article are solely those of the authors and do not necessarily represent those of their affiliated organizations, or those of the publisher, the editors and the reviewers. Any product that may be evaluated in this article, or claim that may be made by its manufacturer, is not guaranteed or endorsed by the publisher.

## Supplementary Material

Supplementary Figure 1 Sensitivity analysis of blood loss **(A)**, operative time **(B)**, and postoperative hospital day **(C)**.

Supplementary Figure 2 Publication bias of postoperative drainage time **(A)**, operative time **(B)**, and postoperative hospital day **(C)**.

Supplementary Table 1 PRISMA 2009 Checklist.

Supplementary Table 2 The strategy of searching.

Supplementary Table 3 GRADE quality assessment of the results through treatment strategy and research design.

## Abbreviations

3D: Three-dimensional
CI: confidence interval
FEV1: Forced Expiratory Volume in the first second
GRADE: Grading of Recommendations Assessment Development and Evaluation
HR: hazard ratio
LC: Lung cancer
LNs: lymph nodes
MD: mean difference
NOS: Newcastle-Ottawa Scale
NSCLC: Non-small cell lung cancer
OP: operation
PRISMA: Preferred Reporting Items for Systematic Reviews and Meta-Analyses
RCT: randomized clinical trial
RR: risk ratio
SMD: standardized mean difference
TNM: Tumor Node Metastasis.
