# Supplementary figures and images for: Preoperative Three-Dimensional Lung Simulation Before Thoracoscopic Anatomical Segmentectomy for Lung Cancer: A Systematic Review and Meta-Analysis
Source: Front Surg. 2022 Mar 31;9:856293. doi: 10.3389/fsurg.2022.856293 (PMC9008247; doi:10.3389/fsurg.2022.856293)

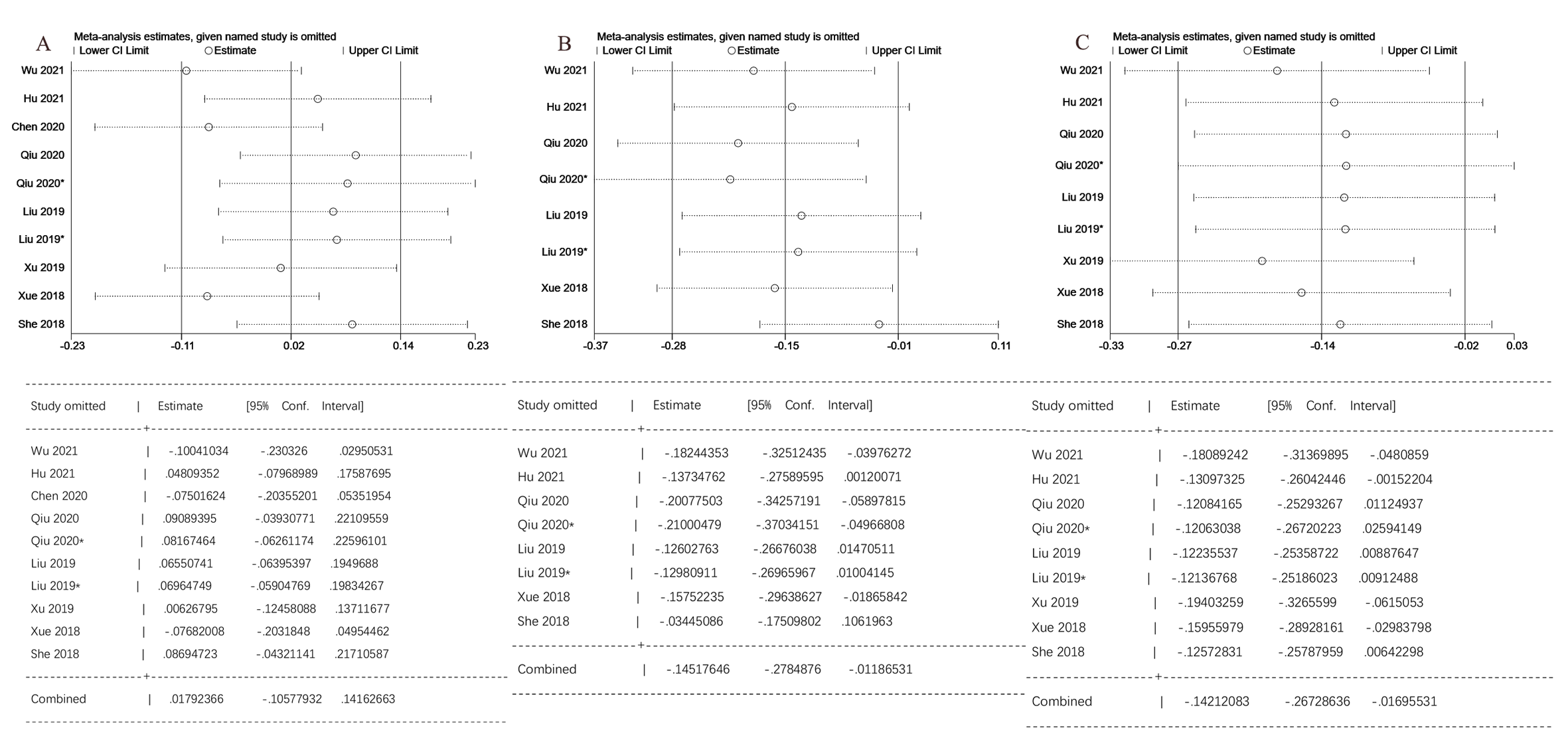

Supplement: Supplementary Figure 1 — Sensitivity analysis of blood loss (A), operative time (B), and postoperative hospital day (C). [file Image_1.TIF]

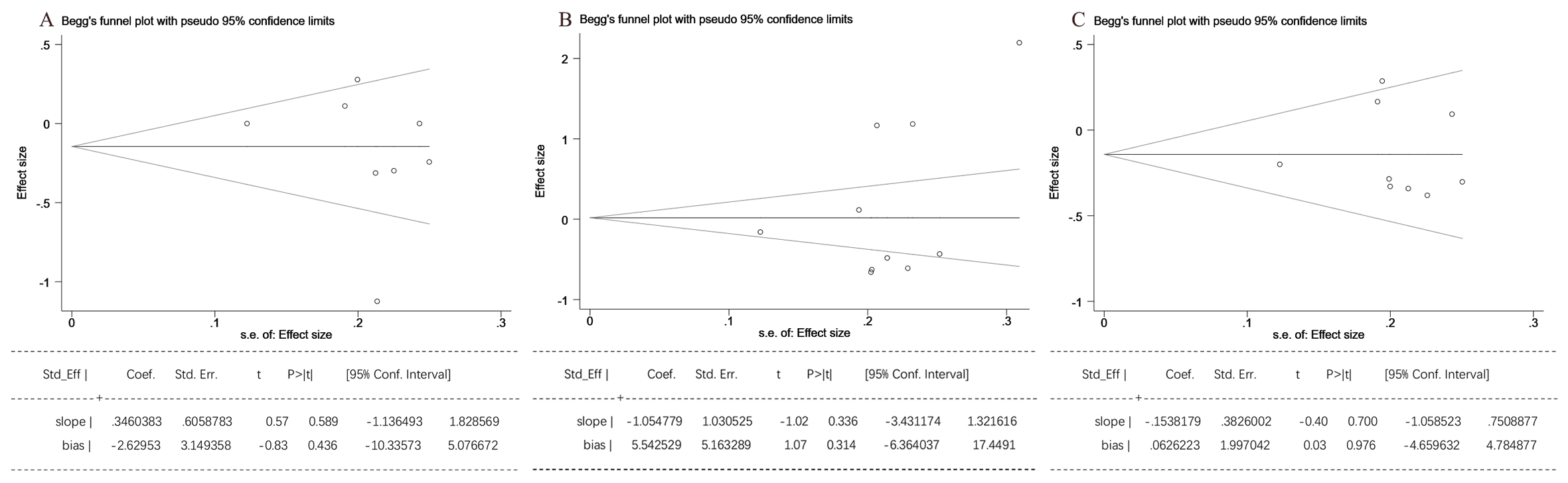

Supplement: Supplementary Figure 2 — Publication bias of postoperative drainage time (A), operative time (B), and postoperative hospital day (C). [file Image_2.TIF]
