# Supplementary material for: Preoperative Three-Dimensional Lung Simulation Before Thoracoscopic Anatomical Segmentectomy for Lung Cancer: A Systematic Review and Meta-Analysis
Source: Front Surg. 2022 Mar 31;9:856293. doi: 10.3389/fsurg.2022.856293 (PMC9008247; doi:10.3389/fsurg.2022.856293)
Supplement: Supplementary Table 2 — The strategy of searching. [file Table_2.DOCX]

**Table S2** The strategy of searching.

| **PubMed**  The database was searched on August 26, 2021, n=414.  Search Strategy:  (Three-dimensional[Title/Abstract]) AND (Mastectomies,Segmental [Title/Abstract] OR Local Excision Mastectomy[Title/Abstract] OR Segmental Mastectomy[Title/Abstract] OR Segmental Mastectomies[Title/Abstract] OR Local Excision Mastectomies[Title/Abstract] OR Mastectomies, Local Excision[Title/Abstract] OR Mastectomy, Local Excision[Title/Abstract] OR Segmentectomy[Title/Abstract] OR Segmentectomies[Title/Abstract] OR Partial Mastectomy[Title/Abstract] OR Mastectomies, Partial[Title/Abstract] OR Mastectomy, Partial[Title/Abstract] OR Partial Mastectomies[Title/Abstract] OR Limited Resection Mastectomy[Title/Abstract] OR Limited Resection Mastectomies[Title/Abstract] OR Mastectomies, Limited Resection[Title/Abstract] OR Mastectomy, Limited Resection[Title/Abstract] OR Lumpectomy[Title/Abstract] OR Lumpectomies[Title/Abstract] OR Breast-Conserving Surgery[Title/Abstract] OR Breast Conserving Surgery[Title/Abstract] OR Breast Quadrantectomy[Title/Abstract] OR Breast Quadrantectomies[Title/Abstract] OR Quadrantectomies, Breast[Title/Abstract] OR Quadrantectomy, Breast[Title/Abstract] OR Surgery, Breast-Conserving[Title/Abstract] OR Breast-Conserving Surgeries[Title/Abstract] OR Surgeries, Breast-Conserving[Title/Abstract] OR Surgery, Breast Conserving[Title/Abstract] OR Breast Conservation Therapy[Title/Abstract] OR Breast Conservation Therapies[Title/Abstract] OR Conservation Therapies, Breast[Title/Abstract] OR Conservation Therapy, Breast[Title/Abstract] OR Breast-Sparing Surgery[Title/Abstract] OR Breast Sparing Surgery[Title/Abstract] OR Breast-Sparing Surgeries[Title/Abstract] OR Surgeries, Breast-Sparing[Title/Abstract] OR Surgery, Breast-Sparing[Title/Abstract]) |
| --- |
| **Web of Science**  The database was searched on August 26, 2021, n=511.  Search Strategy:  1 TOPIC: (“ Three-dimensional”) (543791)  2 TOPIC: (“Mastectomies, Segmental” OR “Local Excision Mastectomy” OR “Segmental Mastectomy” OR “Segmental Mastectomies” OR “Local Excision Mastectomies” OR “Mastectomies, Local Excision” OR “Mastectomy, Local Excision” OR “Segmentectomy” OR “Segmentectomies” OR “Partial Mastectomy” OR “Mastectomies, Partial” OR “Mastectomy, Partial” OR “Partial Mastectomies” OR “Limited Resection Mastectomy” OR “Limited Resection Mastectomies” OR “Mastectomies, Limited Resection” OR “Mastectomy, Limited Resection” OR “Lumpectomy” OR “Lumpectomies” OR “Breast-Conserving Surgery” OR “Breast Conserving Surgery” OR “Breast Quadrantectomy” OR “Breast Quadrantectomies” OR “Quadrantectomies, Breast” OR “Quadrantectomy, Breast” OR “Surgery, Breast-Conserving” OR “Breast-Conserving Surgeries” OR “Surgeries, Breast-Conserving” OR “Surgery, Breast Conserving” OR “Breast Conservation Therapy” OR “Breast Conservation Therapies” OR “Conservation Therapies, Breast” OR “Conservation Therapy, Breast” OR “Breast-Sparing Surgery” OR “Breast Sparing Surgery” OR “Breast-Sparing Surgeries” OR “Surgeries, Breast-Sparing” OR “Surgery, Breast-Sparing”) (22100)  4 #1 AND #2 (511) |
| **EMBASE**  The database was searched on August 26, 2021, n=531.  Search Strategy:  ('Three-dimensional':ti,ab,kw) AND (' Lumpectomy':ti,ab,kw OR 'Mastectomies, Segmental':ti,ab,kw OR 'Segmental Mastectomies':ti,ab,kw OR 'Segmental Mastectomy':ti,ab,kw OR 'Local Excision Mastectomy':ti,ab,kw OR 'Local Excision Mastectomies':ti,ab,kw OR 'Mastectomies, Local Excision':ti,ab,kw OR 'Mastectomy, Local Excision':ti,ab,kw OR 'Segmentectomy':ti,ab,kw OR 'Segmentectomies':ti,ab,kw OR 'Partial Mastectomy':ti,ab,kw OR 'Mastectomies, Partial':ti,ab,kw OR 'Mastectomy, Partial':ti,ab,kw OR 'Partial Mastectomies':ti,ab,kw OR 'Limited Resection Mastectomy':ti,ab,kw OR 'Limited Resection Mastectomies':ti,ab,kw OR 'Mastectomies, Limited Resection':ti,ab,kw OR 'Mastectomy, Limited Resection':ti,ab,kw OR 'Lumpectomies':ti,ab,kw OR 'Breast-Conserving Surgery':ti,ab,kw OR 'Breast Conserving Surgery':ti,ab,kw OR 'Breast Quadrantectomy':ti,ab,kw OR 'Breast Quadrantectomies':ti,ab,kw OR 'Quadrantectomies, Breast':ti,ab,kw OR 'Quadrantectomy, Breast':ti,ab,kw OR 'Surgery, Breast-Conserving':ti,ab,kw OR 'Breast-Conserving Surgeries':ti,ab,kw OR 'Surgeries, Breast-Conserving':ti,ab,kw OR 'Surgery, Breast Conserving':ti,ab,kw OR 'Breast Conservation Therapy':ti,ab,kw OR 'Breast Conservation Therapies':ti,ab,kw OR 'Conservation Therapies, Breast':ti,ab,kw OR 'Conservation Therapy, Breast':ti,ab,kw OR 'Breast-Sparing Surgery':ti,ab,kw OR 'Breast Sparing Surgery':ti,ab,kw OR 'Breast-Sparing Surgeries':ti,ab,kw OR 'Surgeries, Breast-Sparing':ti,ab,kw OR 'Surgery, Breast-Sparing':ti,ab,kw) |
| **Cochrane Library**  The database was searched on August 26, 2021, n=36.  Search Strategy:  (“Three-dimensional”): ti,ab,kw AND (“Mastectomies, Segmental” OR “Local Excision Mastectomy” OR “Segmental Mastectomy” OR “Segmental Mastectomies” OR “Local Excision Mastectomies” OR “Mastectomies, Local Excision” OR “Mastectomy, Local Excision” OR “Segmentectomy” OR “Segmentectomies” OR “Partial Mastectomy” OR “Mastectomies, Partial” OR “Mastectomy, Partial” OR “Partial Mastectomies” OR “Limited Resection Mastectomy” OR “Limited Resection Mastectomies” OR “Mastectomies, Limited Resection” OR “Mastectomy, Limited Resection” OR “Lumpectomy” OR “Lumpectomies” OR “Breast-Conserving Surgery” OR “Breast Conserving Surgery” OR “Breast Quadrantectomy” OR “Breast Quadrantectomies” OR “Quadrantectomies, Breast” OR “Quadrantectomy, Breast” OR “Surgery, Breast-Conserving” OR “Breast-Conserving Surgeries” OR “Surgeries, Breast-Conserving” OR “Surgery, Breast Conserving” OR “Breast Conservation Therapy” OR “Breast Conservation Therapies” OR “Conservation Therapies, Breast” OR “Conservation Therapy, Breast” OR “Breast-Sparing Surgery” OR “Breast Sparing Surgery” OR “Breast-Sparing Surgeries” OR “Surgeries, Breast-Sparing” OR “Surgery, Breast-Sparing”): ti,ab,kw - (Word variations have been searched) |
| **Ovid MEDLINE**  The database was searched on August 26, 2021, n=473.  Search Strategy:  1 Three-dimensional.ab.  2 Mastectomies, Segmental.ab.  3 Segmental Mastectomies.ab.  4 Segmental Mastectomy.ab.  5 Local Excision Mastectomy.ab.  6 Local Excision Mastectomies.ab.  7 Mastectomies, Local Excision.ab.  8 Mastectomy, Local Excision.ab.  9 Segmentectomy.ab.  10 Segmentectomies.ab.  11 Partial Mastectomy.ab.  12 Mastectomies, Partial.ab.  13 Mastectomy, Partial.ab.  14 Partial Mastectomies.ab.  15 Limited Resection Mastectomy.ab.  16 Limited Resection Mastectomies.ab.  17 Mastectomies, Limited Resection.ab.  18 Mastectomy, Limited Resection.ab.  19 Lumpectomy.ab.  20 Lumpectomies.ab.  21 Breast-Conserving Surgery.ab.  22 Breast Conserving Surgery.ab.  23 Breast Quadrantectomy.ab.  24 Breast Quadrantectomies.ab.  25 Quadrantectomies, Breast.ab.  26 Quadrantectomy, Breast.ab.  27 Surgery, Breast-Conserving.ab.  28 Breast-Conserving Surgeries.ab.  29 Surgeries, Breast-Conserving.ab.  30 Surgery, Breast Conserving.ab.  31 Breast Conservation Therapy.ab.  32 Breast Conservation Therapies.ab.  33 Conservation Therapies, Breast.ab.  34 Conservation Therapy, Breast.ab.  35 Breast-Sparing Surgery.ab.  36 Breast Sparing Surgery, Breast.ab.  37 Breast-Sparing Surgeries, Breast.ab.  38 Surgeries, Breast-Sparing.ab.  39 Surgery, Breast-Sparing.ab.  40 or/2-39[Segmentectomy]  42 1 and 40 (473) |
| **ScienceDirect**  The database was searched on August 26, 2021, n=25.  Search Strategy:  Title, abstract, keywords: ((“Three-dimensional”) and (“Mastectomies, Segmental” OR “Local Excision Mastectomy” OR “Segmental Mastectomy” OR “Segmental Mastectomies” OR “Local Excision Mastectomies” OR “Mastectomies, Local Excision” OR “Mastectomy, Local Excision” OR “Segmentectomy” OR “Segmentectomies” OR “Partial Mastectomy” OR “Mastectomies, Partial” OR “Mastectomy, Partial” OR “Partial Mastectomies” OR “Limited Resection Mastectomy” OR “Limited Resection Mastectomies” OR “Mastectomies, Limited Resection” OR “Mastectomy, Limited Resection” OR “Lumpectomy” OR “Lumpectomies” OR “Breast-Conserving Surgery” OR “Breast Conserving Surgery” OR “Breast Quadrantectomy” OR “Breast Quadrantectomies” OR “Quadrantectomies, Breast” OR “Quadrantectomy, Breast” OR “Surgery, Breast-Conserving” OR “Breast-Conserving Surgeries” OR “Surgeries, Breast-Conserving” OR “Surgery, Breast Conserving” OR “Breast Conservation Therapy” OR “Breast Conservation Therapies” OR “Conservation Therapies, Breast” OR “Conservation Therapy, Breast” OR “Breast-Sparing Surgery” OR “Breast Sparing Surgery” OR “Breast-Sparing Surgeries” OR “Surgeries, Breast-Sparing” OR “Surgery, Breast-Sparing”)) |
| **Scopus**  The database was searched on August 26, 2021, n=5393.  Search Strategy:  TITLE-ABS-KEY ((“Three-dimensional”) and (“Mastectomies, Segmental” OR “Local Excision Mastectomy” OR “Segmental Mastectomy” OR “Segmental Mastectomies” OR “Local Excision Mastectomies” OR “Mastectomies, Local Excision” OR “Mastectomy, Local Excision” OR “Segmentectomy” OR “Segmentectomies” OR “Partial Mastectomy” OR “Mastectomies, Partial” OR “Mastectomy, Partial” OR “Partial Mastectomies” OR “Limited Resection Mastectomy” OR “Limited Resection Mastectomies” OR “Mastectomies, Limited Resection” OR “Mastectomy, Limited Resection” OR “Lumpectomy” OR “Lumpectomies” OR “Breast-Conserving Surgery” OR “Breast Conserving Surgery” OR “Breast Quadrantectomy” OR “Breast Quadrantectomies” OR “Quadrantectomies, Breast” OR “Quadrantectomy, Breast” OR “Surgery, Breast-Conserving” OR “Breast-Conserving Surgeries” OR “Surgeries, Breast-Conserving” OR “Surgery, Breast Conserving” OR “Breast Conservation Therapy” OR “Breast Conservation Therapies” OR “Conservation Therapies, Breast” OR “Conservation Therapy, Breast” OR “Breast-Sparing Surgery” OR “Breast Sparing Surgery” OR “Breast-Sparing Surgeries” OR “Surgeries, Breast-Sparing” OR “Surgery, Breast-Sparing”)) |

**Note:** The combined text and medical subject heading (MeSH) terms used were: “Three-dimensional” and “Segmentectomy”.
