# Supplementary material for: Preoperative Three-Dimensional Lung Simulation Before Thoracoscopic Anatomical Segmentectomy for Lung Cancer: A Systematic Review and Meta-Analysis
Source: Front Surg. 2022 Mar 31;9:856293. doi: 10.3389/fsurg.2022.856293 (PMC9008247; doi:10.3389/fsurg.2022.856293)
Supplement: Supplementary Table 3 — GRADE quality assessment of the results through treatment strategy and research design. [file Table_3.DOCX]

**Table S3** GRADE quality assessment of the results through treatment strategy and research design.

| **Primary outcomes** | **No. of Studies** | **No. of Participants** | | | **Differences (95%CI) ^a^** | | **Quality Assessment** | | | | | | | | | | **Quality** | |  |  |  |
| --- | --- | --- | --- | --- | --- | --- | --- | --- | --- | --- | --- | --- | --- | --- | --- | --- | --- | --- | --- | --- | --- |
|  |  | **3D group** | **Non-3D group** | |  |  | **Risk of Bias^b^** | | **Inconsistency** | | **Indirectness** | | **Imprecision** | | **Publication Bias^c^** | |  |  |  |  |  |
|  |  |  |  |  |  |  |  |  |  |  |  |  |  |  |  |  |  |  |  | | |
| **Preoperative indications** | |  |  | |  | |  | |  | |  | |  | |  | |  | |  | |  |
| Mean nodule size | 4 | 299 | 256 | | -0.99[-1.58,-0.40] | | Low | | No inconsistency | | No indirectness | | No imprecision | | Unlikely | | Low | |  | | |
| Active smokers | 5 | 80/396 | 51/294 | | 1.24[0.92, 1.68] | | Low | | Very Serious (-2) | | No indirectness | | No imprecision | | Unlikely | | Very Low | |  | | |
| FEV1 %pred | 3 | 294 | 205 | | -1.55[-4.32, 1.21] | | Low | | No inconsistency | | No indirectness | | No imprecision | | Unlikely | | Low | |  | | |
| **Comorbidity** |  |  |  | |  | |  | |  | |  | |  | |  | |  | |  | | |
| Hypertension | 4 | 65/320 | 55/272 | | 1.01 [0.74-1.40] | | Low | | No inconsistency | | No indirectness | | No imprecision | | Unlikely | | Low | |  | | |
| Diabetes mellitus | 4 | 33/320 | 23/272 | | 1.29 [0.77-2.15] | | Low | | No inconsistency | | No indirectness | | No imprecision | | Unlikely | | Low | |  | | |
| COPD | 3 | 13/284 | 14/240 | | 0.81 [0.40-1.67] | | Low | | No inconsistency | | No indirectness | | No imprecision | | Unlikely | | Low | |  | | |
| Heart diseases | 3 | 13/269 | 11/221 | | 0.97[0.44-2.12] | | Low | | No inconsistency | | No indirectness | | No imprecision | | Unlikely | | Low | |  | | |
| Arrhythmia | 2 | 4/213 | 6/187 | | 0.61 [0.18-2.12] | | Low | | No inconsistency | | No indirectness | | No imprecision | | Unlikely | | Low | |  | | |
| Non-thoracic surgery history | 1 | 3/36 | 2/32 | | 1.33 [0.24-7.48] | | Low | | No inconsistency | | No indirectness | | No imprecision | | Unlikely | | Low | |  | | |
| **Intraoperative outcomes** | |  |  | |  | |  | |  | |  | |  | |  | |  | |  | |  |
| Blood loss | 8 | 552 | 437 | | -16.21[-24.95,-7.47] | | Low | | No inconsistency | | No indirectness | | No imprecision | | Unlikely | | Low | |  | | |
| Operative time | 8 | 552 | 437 | | -1.97[-14.48, 10.54] | | Low | | No inconsistency | | No indirectness | | No imprecision | | Unlikely | | Low | |  | | |
| Conversion* | 3 | 122 | 91 | | 0.12[0.03, 0.48] | | Low | | No inconsistency | | No indirectness | | Serious (-1) | | Unlikely | | Very Low | |  | | |
| Number of resected LNs | 2 | 106 | 106 | | 0.94[-1.22, 3.09] | | Low | | No inconsistency | | No indirectness | | No imprecision | | Unlikely | | Low | |  | | |
| **Hospitalization and follow up outcomes** | |  |  | |  | |  | |  | |  | |  | |  | |  | |  | |  |
| Postoperative hospital stay | 7 | 501 | 399 | | -0.25[-0.46,-0.04] | | Low | | No inconsistency | | No indirectness | | No imprecision | | Unlikely | | Low | |  | | |
| Postoperative drainage time | 6 | 405 | 362 | | -0.30[-0.76, 0.17] | | Low | | Very Serious (-2) | | No indirectness | | No imprecision | | Unlikely | | Very Low | |  | | |
| Total complications | 8 | 552 | 437 | | 0.64 [0.44, 0.93] | | Low | | No inconsistency | | No indirectness | | No imprecision | | Unlikely | | Low | |  | | |
| Postoperative drainage volume | 3 | 309 | 224 | | -22.00[-190.18,146.19] | | Very Serious (-2) | | No inconsistency | | No indirectness | | No imprecision | | Unlikely | | Very Low | |  | | |
| Post-op FEV1 | 1 | 36 | 32 | | 1.00 [0.78, 1.28] | | Low | | No inconsistency | | No indirectness | | No imprecision | | Unlikely | | Low | |  | | |
| **Postoperative complication** | | | |  | |  | |  | |  | |  | |  | |  | |  | |  |  |
| Pulmonary air leakage | 8 | 15/552 | 24/437 | | 0.53[0.28-1.01] | | Low | | No inconsistency | | No indirectness | | No imprecision | | Unlikely | | Low | |  | | |
| Pneumonia | 6 | 7/405 | 8/362 | | 0.83[0.33-2.08] | | Low | | No inconsistency | | No indirectness | | No imprecision | | Unlikely | | Low | |  | | |
| Atelectasis | 5 | 6/371 | 7/310 | | 0.72[0.26-2.01] | | Low | | No inconsistency | | No indirectness | | No imprecision | | Unlikely | | Low | |  | | |
| Hemoptysis | 4 | 3/207 | 16/194 | | 0.19[0.06-0.58] | | Low | | Very Serious (-2) | | No indirectness | | No imprecision | | Unlikely | | Very Low | |  | | |
| Arrhythmia | 3 | 7/132 | 11/124 | | 0.67[0.28-1.60] | | Low | | No inconsistency | | No indirectness | | No imprecision | | Unlikely | | Low | |  | | |
| Atrial fibrillation | 2 | 2/91 | 0/87 | | 2.83[0.30-26.68] | | Low | | No inconsistency | | No indirectness | | No imprecision | | Unlikely | | Low | |  | | |
| Cerebral infarction | 2 | 2/147 | 1/75 | | 0.91[0.12-7.17] | | Low | | No inconsistency | | No indirectness | | No imprecision | | Unlikely | | Low | |  | | |
| Pulmonary infection | 2 | 3/147 | 2/75 | | 0.75[0.13-4.10] | | Low | | No inconsistency | | No indirectness | | Serious (-1) | | Unlikely | | Very low | |  | | |
| Liquid pneumothorax | 1 | 1/96 | 0/37 | | 1.18[0.05-28.22] | | Low | | No inconsistency | | No indirectness | | No imprecision | | Unlikely | | Low | |  | | |
| Postoperative hemothorax | 1 | 1/96 | 0/37 | | 1.18[0.05-28.22] | | Low | | No inconsistency | | No indirectness | | No imprecision | | Unlikely | | Low | |  | | |
| Pleural effusion | 1 | 1/96 | 0/37 | | 1.18[0.05-28.22] | | Low | | No inconsistency | | No indirectness | | No imprecision | | Unlikely | | Low | |  | | |

**Abbreviations:** CI: confidence interval. COPD: chronic obstructive pulmonary disease. LNs: lymph nodes. Post-op FEV1: postoperative Forced Expiratory Volume in the first second. Conversion*: conversion from segmentectomy to thoracotomy or lobectomy.

^a^ Differences: risk ratios (RR) for active smokers, Hypertension, Diabetes mellitus, COPD, heart diseases, arrhythmia, cancer history, non-thoracic surgery history, pulmonary air leakage, pneumonia, atelectasis, hemoptysis, atrial fibrillation, cerebral infarction, pulmonary infection, liquid pneumothorax, postoperative hemothorax, pleural effusion; mean difference (MD) for mean nodule size, FEV1%pred, blood loss, operative time, resected LNs, conversion, postoperative hospital stay, postoperative drainage time, total complications, postoperative drainage volume.

^b^ Risk of bias assessed using the Newcastle-Ottawa Scale (NOS) for non-randomized studies and Jadad scale for randomized controlled trials.

^c^ Publication bias was assessed by Egger’s and Begg’s tests.
